# Evaluate the Prognostic Value of serumγKlotho on Long‐Term Prognosis of Patients With Multivessel Coronary Artery Disease and Establish a Prognostic Model

**DOI:** 10.1002/clc.70393

**Published:** 2026-06-24

**Authors:** Tuersunjiang Naman, Yu‐ting Zhang, Ayinuer Maihemuti, Hui Cheng, Wen‐bo Fu, Xiao‐lin Yu, Zhao Wang, Yi‐ning Yang, Zi‐tong Guo

**Affiliations:** ^1^ Department of Cardiology, People's Hospital of Xinjiang Uygur Autonomous Region Affiliated Hospital of Xinjiang Second Medical College Urumqi Xinjiang China

**Keywords:** long‐term prognosis, Multivessel disease, predictive model, serum γKlotho

## Abstract

**Objective:**

This study was aimed at analyzing the correlation of serum γKlotho with the long‐term prognosis of multivessel coronary artery disease (MVD) and develop a predictive model to predict an accurate long‐term prognosis.

**Methods:**

We enrolled 969 MVD patients and classified them into three groups: training (*n* = 552), internal validation (*n* = 224), and external validation (*n* = 193) groups, respectively. The training group data helped in establishing a prognostic model. Univariable Cox regression analyses were conducted using serum and clinical γKlotho levels. Thereafter, the least absolute shrinkage and selection operator (LASSO) regression model was utilized for optimizing feature selection. Additionally, we constructed a prognosis prediction nomogram with multivariate Cox regression results by incorporating features screened with the LASSO model. Moreover, the as‐constructed prognosis model's discriminability, consistency, and clinical usefulness were assessed by receiver operating characteristic (ROC) curve, C‐index, decision curve analysis (DCA), and calibration plot analysis, respectively.

**Result:**

Included in our prognosis prediction nomogram, the predictors of the long‐term prognosis of MVD patients were age, BMI, diabetes mellitus (DM), antiplatelets, LDL‐C, LVDD, and serum γKlotho level. Consequently, this nomogram displayed high discriminability, according to ROC and C‐index analyses. Moreover, according to the calibration plot, the nomogram's probabilities exhibited high consistency with the actual levels. Based on DCA, this nomogram displayed good clinical usefulness.

**Conclusion:**

Higher serum γKlotho levels correlated with the poor prognosis of MVD patients, our predictive model exhibited better predictive ability and clinical usefulness.

## Graphical abstract

Higher serum γKlotho level correlated with the poor prognosis of MVD patients, our predictive model exhibited better predictive ability and clinical usefulness.

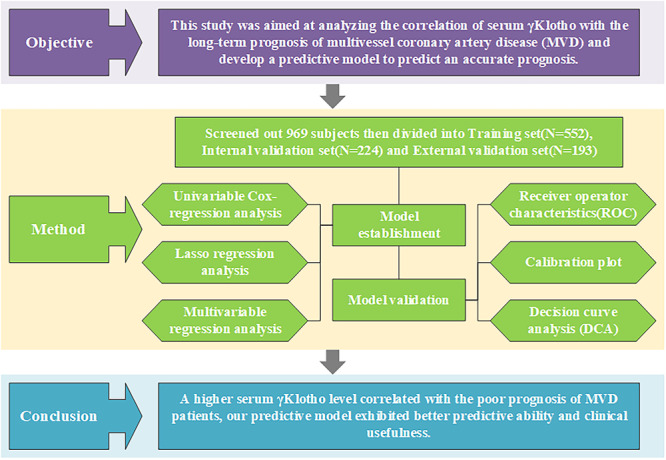

## 1 Introduction

Cardiovascular disease (CVD) is the main reason for death globally. Coronary heart disease (CHD) occupies a relatively large proportion of all CVDs; the incidence of CHD was 7.2% in accordance with the global demographic disease statistics [1].

The growing proportion of main adverse cardiovascular events (MACEs) caused by CHD results in heavy physical, health, and financial burdens on patients and their families [2, 3]. Multivessel coronary artery disease (MVD) exhibits a high prevalence rate [4], induces more adverse cardiovascular events, and reflects a poorer prognosis than other types of CHD patients [5].

There are various revascularization strategies to manage MVD, include interventional percutaneous coronary intervention (PCI) or coronary artery bypass grafting (CABG) revascularization [6]. Nevertheless, some MVD patients are not treated with PCI or CABG because they are unwilling to undergo PCI or CABG.

Current clinical practice guidelines [7, 8] indicate that MVD patients who are not treated with PCI or CABG exhibit enhanced major adverse cardiovascular events (MACEs) relative to patients treated with CABG or PCI.

Thus, it is crucial to investigate risk stratification markers in MVD patients without PCI or CABG and perform an accurate risk stratification to develop relevant therapeutic strategies, enhance the patient's safety and quality of life, as well as assess the need for PCI or CABG treatments.

Klotho, first discovered in 1997, is a new biomarker correlated with human health and various disease status as well as human longevity [9]. The Klotho family includes three members encoded by three genes [10, 11]: α‐Klotho, β‐Klotho, and γ‐Klotho. In previous study reported that:α‐Klotho and β‐Klotho are correlated with the risk of CVD [12, 13] and diabetes [14]. As to γKlotho, previous research reported that: γKlotho is correlated with breast cancer [15], bladder cancer [16], and prostate cancer [17], but lack of research on γKlotho and CVD. In our previous study, serum γKlotho level represented the risk factor related to the incidence of CHD [18], but its impact on the prognosis of CHD patients, especially in MVD patients, is unclear.

Thus, this study aimed to analyze the correlation of serum γKlotho level with the long‐term prognosis of MVD, as well as develop a clinical nomogram to accurately predict the prognosis of MVD.

## 2 Materials and Methods

### 2.1 Study Subjects and Design

**Figure 1:**
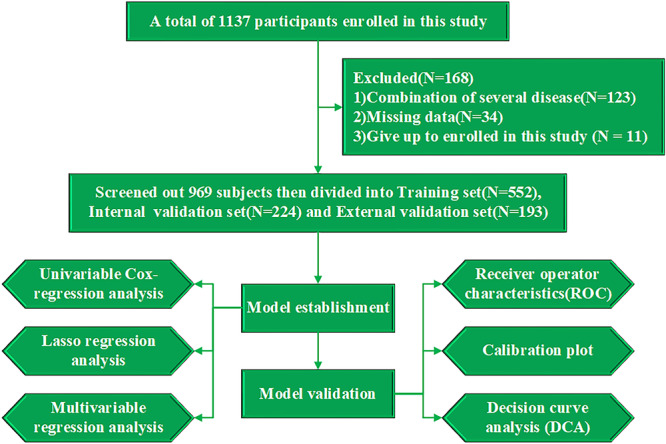
The study's flowchart.

A total of 1137 patients were recruited at the People's Hospital of Xinjiang Uyghur Autonomous Region (*n* = 944) and at the First affiliated hospital of Xinjiang medical university (*n* = 193, used to external validation) from January 2013 to December 2018. In line with our predetermined eligibility standards, we finally enrolled 969 patients in our study. All patients offered informed consent for participation (Figure 1).

The inclusion criteria were: patients whose coronary angiography or computed tomography angiography (CTA) revealed ≥ 50% luminal stenosis in the left main coronary artery or at least two major epicardial arteries but not received PCI or CABG.

The exclusion criteria were: those having acute decompensated heart failure (HF), patients with unstable hemodynamics, liver/kidney/autoimmune/hematological disorders, cachexia, non‐cardiac conditions with a < 1‐year life expectancy, and those refusing to participate in the study.

### 2.2 Blood Sample Collection and Laboratory Tests

Blood was collected from all MVD cases and sent to laboratory of People's Hospital of Xinjiang Uyghur Autonomous Region to test laboratory parameters like platelet (PLT), white blood cell (WBC) counts (examined by Electrical impedance analysis [EIA]), hemoglobin level (assessed by chemical colorimetry method), creatinine (CR), blood urea nitrogen (BUN), triglyceride (TG), total cholesterol (TC), high‐ and low‐density lipoprotein‐cholesterol (HDL‐C and LDL‐C, detected by chemiluminescence method).

### 2.3 Test the Serum γKlotho Level

After taking samples, blood was centrifuged (1500 rpm, 10 min) for separating plasma and blood cells in an Eppendorf high‐speed centrifuge with anticoagulant ethylene diamine tetraacetic acid (EDTA). Additionally, plasma was kept at −80°C until the evaluation of the serum γKlotho level.

γKlotho ELISA kit (mlbio, Shanghai, China) was utilized to examine serum γKlotho level in a blinded manner; the examiners did not know the participants' clinical data. Moreover, 10% of duplicate samples were set to analyze genotyping quality.

### 2.4 Cardiovascular Risk Factor Identification

Body mass index (BMI) was determined by dividing the body weight (kg) by the body height squared (m). Smokers comprised individuals who had ever smoked for > 6 months or in the past 6 months. Drinkers referred to those who consumed >100 g alcoholic beverages every week in the last month. Hypertension was defined in accordance with the 2011 New NICE guidelines for hypertension [19], including systolic blood pressure (SBP) ≥ 140 mmHg, diastolic blood pressure (DBP) ≥ 90 mmHg, or using antihypertensive medications within the past 2 weeks. Diabetes mellitus (DM) implied a fasting plasma glucose level ≥ 7.0 mmol/L (126 mg/dL), a glucose level being 11.1 mmol/L (200 mg/dL) 2 h post‐oral glucose administration (75 g), using anti‐diabetic medications, or having a DM history.

### 2.5 Follow‐up Endpoints

Our endpoint included the MACEs after diagnosis with MVD [20], such as cardiogenic death, non‐fatal myocardial infarction (MI), stroke, and ACS‐triggered unplanned revascularization. Non‐fatal MI comprised the ischemic event meeting the ESC/American College of Cardiology criteria for MI, which could be distinguished from baseline ACS post‐admission [21]. Stroke is referred to as an ischemic event according to the European Stroke Organization guidelines [22]. ACS‐triggered repeat revascularization implies a second unexpected PCI or CABG carried out in an emergency due to acute ischemic symptoms [23]. The duration time between being diagnosed with MVD for the first time to having MACEs for the first time was recorded. All participants and their families were contacted through telephone calls. Telephone interviews were performed with patients or their family members at 3, 6, 12, 24, and 60 months after diagnosed with MVF to obtain the prognostic data. Follow‐ups were carried out by trained staff, while data were imported by three experienced investigators to guarantee data quality. Clinicians who were trained in acquiring systemic data and confirming events were responsible for follow‐ups.

### 2.6 Statistical Analysis

R 4.2.1 and SPSS 27.0 software were utilized in statistical analyses. All participants were categorized into two groups: training and validation groups. Training group data were classified into two groups: non‐MACEs and MACEs groups, respectively. Using univariable Cox regression analysis, we screened out the variables with *p* < 0.2 and performed LASSO regression analysis by utilizing these variables; features showing non‐zero coefficients were chosen [24]. Multivariable Cox regression analysis was performed for developing a prognosis prediction model using LASSO model‐selected variables. The threshold of total points was an intersection point to classify the subjects in high‐ and low‐risk groups, and the scatter plot of the corresponding MACE time for diverse samples was created. The accumulated MACE curves for high‐ and low‐risk patients were plotted with Kaplan–Meier (KM) curves, whereas the *p* value was determined. Meanwhile, the cut‐off value of the serum γKlotho level was used to divide patients into high‐ or low‐serum γKlotho level groups, respectively. Subsequently, we plotted the accumulated MACE curves for both groups.

### 2.7 Model Validation

Through internal and external validation, our constructed nomogram was assessed for discriminability (according to AUC value and C‐index), clinical usefulness (using the DCA curve), and consistency (using the calibration plot). Firstly, ROC curves were plotted, and the C‐index was calculated for identifying discrimination ability; the value data approaching 1 indicated superior model performance. [25] Secondly, we drew calibration plots [26] for determining the consistency between predicted and actual probabilities. As revealed by the 45°diagonal line, our constructed model achieved a high prediction performance of disease prevalence. Thirdly, we conducted DCA [27] to analyze the model's clinical usefulness according to the net benefits upon diverse threshold probabilities.

## 3 Results

We included 969 MVD patients, of which 506 were non‐MACEs patients at the 60‐month follow‐up, 58 were lost to follow‐up and 405 exhibited MACEs, comprising 99 (9.9%) cardiogenic death, 135 (13.9%) non‐fatal MI, 91 (9.4%) ACS‐driven unplanned revascularization, and 83 (8.6%) stroke cases, respectively.

All subjects were classified into training set (*n* = 552), internal validation set (*n* = 224) set and external validation set (*n* = 193, subjects who hospitalized in the First Affiliated Hospital of Xinjiang Medical University), respectively. Additionally, the training set was classified into two groups as per the 60‐month follow‐up results: non‐MACEs and MACEs. Univariate Cox regression was conducted by incorporating basic clinical features and serumγKlotho level of non‐MACEs and MACEs groups, respectively. Consequently, age, DM, hemoglobin, LDL‐C, serum albumin, LVED, γKlotho level, and antiplatelets were significantly different (*p* < 0.05). Moreover, sex, smoking, alcohol consumption, hypertension, atrial and ventricular arrhythmia, WBC, PLT, TC, TG, HDL‐C, C‐reactive protein (CRP), Aspartate Aminotransferase (AST), Alanine Aminotransferase (ALT), BUN, serum chlorine, total bilirubin, BNP, ejection fraction, LVDD, statin, ACEI/ARB, and β‐blockers did not show any significant difference (*p* > 0.05, Table 1).

**Table 1:** Univariate Cox regression results.

Variable	*β*	SE	*χ* ^2^	HR (95% CI)	*p* value
Age	0.024	0.006	18.292	1.025 (1.013–1.036)	< 0.001
Sex					
Male				Reference	
Female	0.267	0.139	3.659	1.306 (0.993–1.716)	0.056
Smoking	0.246	0.139	3.138	1.279 (0.974–1.678)	0.076
Alcohol	0.263	0.146	3.265	1.301 (0.978–1.731)	0.071
Hypertension	0.250	0.145	2.968	1.285 (0.966–1.708)	0.085
Diabetes	0.631	0.151	17.367	1.880 (1.397–2.530)	< 0.001
Atrial arrhythmia	0.038	0.164	0.054	1.039 (0.753–1.434)	0.816
Ventricular arrhythmia	0.131	0.156	0.710	1.141 (0.840–1.549)	0.399
VBC	0.047	0.042	1.249	1.049 (0.965–1.140)	0.264
PLT	−0.002	0.001	1.402	0.998 (0.996–1.001)	0.236
HGB	−0.008	0.004	3.920	0.992 (0.985–1.000)	0.048
TC	0.017	0.064	0.069	1.017 (0.898–1.152)	0.792
TG	0.034	0.052	0.419	1.035 (0.934–1.146)	0.517
HDL	−0.204	0.259	0.618	0.816 (0.491–1.355)	0.432
LDL	0.195	0.057	11.516	1.215 (1.086–1.360)	0.001
CRP	0.005	0.007	0.613	1.005 (0.992–1.018)	0.434
AST	−0.001	0.003	0.160	0.999 (0.993–1.004)	0.689
ALT	−0.001	0.001	0.245	0.999 (0.996–1.002)	0.621
BUN	0.012	0.021	0.321	1.012 (0.971–1.054)	0.571
Crea	0.003	0.001	2.902	1.003 (1.000–1.005)	0.088
Total‐bolorubin	0.005	0.006	0.573	1.005 (0.992–1.018)	0.449
Albumin	−0.064	0.019	11.405	0.938 (0.904–0.974)	0.001
BNP × 10^3^	0.651	0.364	3.197	1.918 (0.939–3.918)	0.074
Ejection fraction	0.01	0.006	3.005	1.010 (0.999–1.022)	0.083
LVDD	0.022	0.007	11.118	1.022 (1.009–1.035)	0.001
Serum γKlotho	0.022	0.006	14.225	1.023 (1.011–1.034)	< 0.001
Antiplatelet	−0.944	0.154	37.626	0.389 (0.288–0.526)	< 0.001
Statin	−0.053	0.217	0.060	0.948 (0.620–1.451)	0.807
ACEI/ARB	−0.271	0.148	3.341	0.763 (0.571–1.020)	0.068
β Blocker	−0.203	0.155	1.708	0.816 (0.602–1.107)	0.191

### 3.1 Clinical Feature Selection

**Figure 2:**
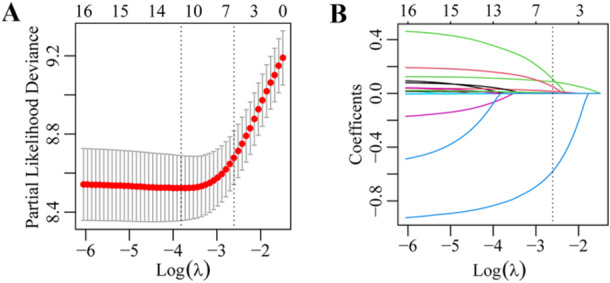
Features are selected with the LASSO model. (A) The tuning parameter (λ) was chosen in the LASSO model with minimum criteria through 10‐fold cross‐validation. Partial likelihood deviance versus log(λ) was plotted. Considering the minimum criteria and its one standard error (1‐SE), we drew dotted vertical lines at optimum values. Thus, λ = 0.074 was chosen (1‐SE criteria) after 10‐fold cross‐validation. (B) LASSO coefficients for 17 features. The coefficient profile versus log (λ) sequence was plotted. A vertical line was plotted at values chosen upon 10‐fold cross‐validation, where optimum λ produced seven non‐zero coefficients.

According to univariate Cox regression results, 17 features (*p* < 0.2) were included in LASSO regression. Through analyzing the 522 study participants in the training group, 17 variables were reduced to 7 that had non‐zero coefficients (Figure 2A,B).

### 3.2 The Prognosis Prediction Model's Construction

Through multivariate Cox regression on seven variables selected post‐LASSO analysis, a prognosis prediction model was constructed (Table 2).

**Table 2:** Multivariable Cox regression results.

Variable	*β*	SE	HR (95% CI)	*p* value
Age	0.039	0.006	1.039 (1.027–1.052)	< 0.001
MBI	0.122	0.015	1.129 (1.096–1.164)	< 0.001
Diabetes				
No			Reference	
Yes	0.467	0.154	1.595 (1.18–2.157)	0.002
LDL‐C	0.207	0.06	1.231 (1.094–1.385)	0.001
LVDD	0.018	0.007	1.018 (1.004–1.033)	0.013
Antiplatelet				
No			Reference	
Yes	−0.911	0.156	0.402 (0.296–0.546)	< 0.001
Serum γKlotho	0.017	0.006	1.017 (1.005–1.029)	0.005

**Figure 3:**
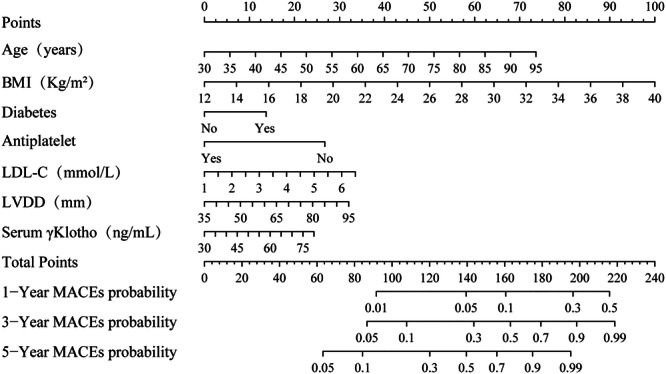
Our nomogram was used to evaluate the MACE rate in MVD patients. Our constructed nomogram included age, BMI, DM, antiplatelets, LDL‐C, Left ventricular end diastolic diameter (LVDD), and Serum γKlotho level.

As suggested, our visualized prognosis prediction model included the independent risk factors having MACEs of MVD, such as age, BMI, DM, LDL‐C, LVDD, antiplatelets, and serum γKlotho level (Figure 3).

### 3.3 Model Verification

**Figure 4:**
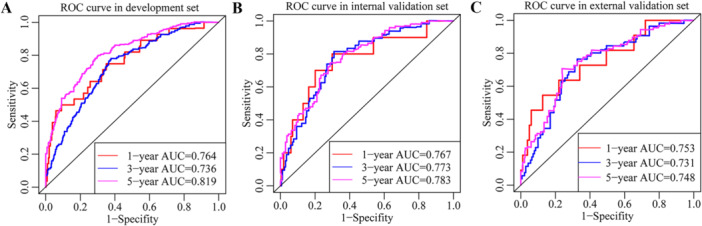
1‐, 2‐ and 3‐year ROC curves and corresponding AUC values of training (A) internal validation (B), and External validation (C) sets.

The prognostic model was verified based on discriminability, consistency between predicted and measured probabilities, and clinical usefulness. In line with the AUC value, our constructed nomogram exhibited enhanced discrimination ability in three datasets (Figures 4A–C).

The 1‐, 2‐, and 3‐year AUCs of the training set were 0.764, 0.736, 0.819, while those of the internal validation set were 0.767, 0.773, 0.783, and 0.753, 0.731, 0.748 in the external validation set, respectively. Besides, C‐index levels were 0.736 (0.702–0.769) in the training set, 0.729 (0.680–0.778) in the internal validation set, and 0.706 (0.649−0.763) in the external validation set. This result suggested that our model possessed good discrimination ability.

**Figure 5:**
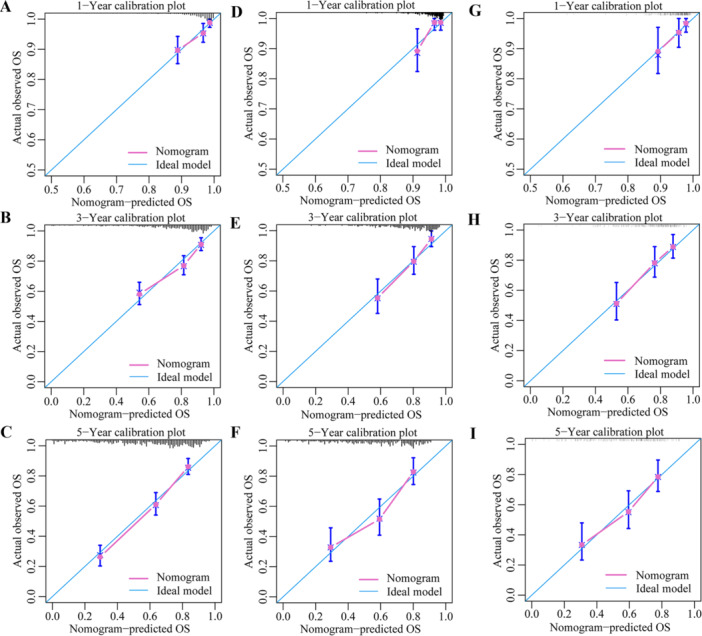
Nomogram calibration plots: 1‐ (A), 3‐ (B), and 5‐ (C) calibration plots of the training set. The 1‐year (D), 3‐year (E), and 5‐year (F) calibration plots in the internal validation set. The 1‐year (G), 3‐year (H), and 5‐year (I) calibration plots in the External validation set. The diagonal blue line represents a perfect prediction by an ideal model. The pink line represents the nomogram's performance conducted by the bootstrap method, of which a closer fit to the diagonal blue line represents a better prediction.

The 1‐, 3‐, and 5‐year calibration plots for the two sets were drawn, which suggested that this prognostic model displayed an enhanced consistency of predicted probabilities versus observed situations (Figure 5).

**Figure 6:**
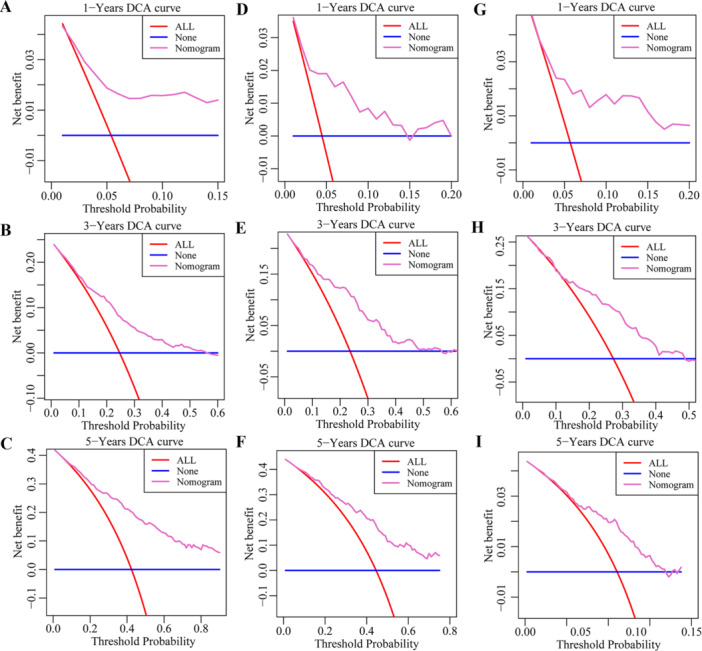
1‐, 3‐, and 5‐year DCAs of the nomogram for training (A–C), Internal validation (D–F) sets and External validation (G–I) set.

And for evaluating the model's clinical usefulness, we conducted DCA (Figure 6).

According to DCA results of the training and internal/external validation sets, the established nomogram was used for predicting the long‐term prognosis, yielding more net benefits than those of “treat‐none” and “treat‐all” strategies. This demonstrated that our model had favorable clinical utility.

### 3.4 Patient Follow‐up

**Figure 7:**
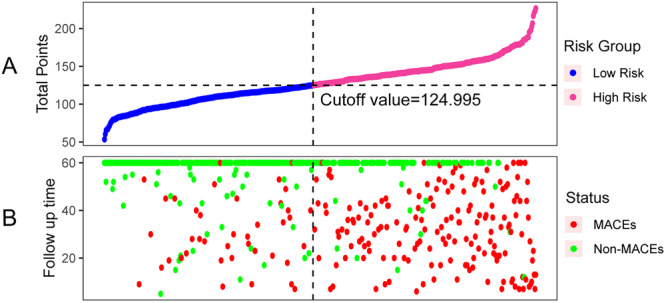
Patients' classification and distribution based on risk and status. (A) Patient classification into high‐ or low‐risk groups based on the threshold nomoscore. (B) MACEs status in high‐ and low‐risk individuals.

We calculated the cutoff value (124.995) of total points and used it as the intersection point to categorize participants into high‐ or low‐risk groups, respectively (Figure 7A). Sensitivity, specificity, positive/negative predictive values (PPV/NPV), and accuracy of 79.97%, 69.72%, 66.02%/82.55%, and 74.06% were obtained, respectively. Besides, the distribution of survival status in both risk groups was presented (Figure 7B).

**Figure 8:**
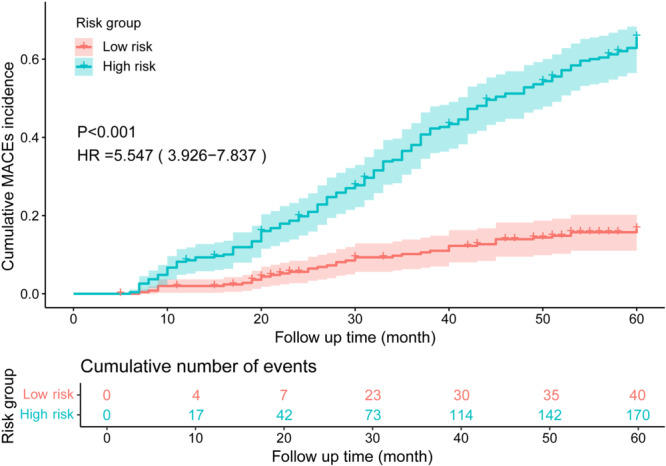
Survival curves for two risk groups.

The KM survival curve was constructed for high‐ and low‐risk groups (Figure 8). Significant differences were observed between the two groups (*p* < 0.001; HR = 5.547; 95% CI: 3.926−7.837).

**Figure 9:**
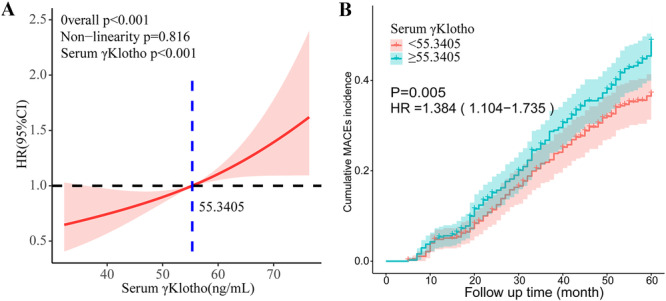
(A) An RCS displaying the relation between serum γKlotho level and long‐term prognosis of MVD patients. (B) Survival curves of high‐ and low serum γKlotho level groups.

Based on our results, serum γKlotho level was a novel biomarker for predicting the long‐term prognosis of MVD patients. For evaluating the connection between serum γKlotho level and long‐term prognosis in MVD patients, we performed restricted cubic spline (RCS, Figure 9A). MACE incidence in MVD patients was positively (*p*_overall_ < 0.001) and linearly (*p*_non‐linearity_ = 0.816) associated with serum γKlotho level. Serum γKlotho level is also positively correlated with the poor prognosis of MVD patients. We calculated the cut‐off value of serum γKlotho level (55.3405 ng/mL) as an intersection point to classify all participants into high‐ or low‐serum γKlotho level groups, respectively. Subsequently, we created a KM survival curve for both groups (Figure 9B); there were significant differences in long‐term prognosis between the two groups (*p* < 0.001; HR = 1.384; 95% CI: 1.104–1.735).

## 4 Discussion

Although there are several traditional risk factors affecting the prognosis of MVD, like age, BMI, dyslipidemia, and so forth, there are various unknown factors that impact the prognosis of MVD, as it is a multifactorial disease. Thus, there is a need to investigate such parameters, explore new risk factors, and establish a risk assessment system.

In our study, we found that age, BMI, DM, antiplatelets, LDL‐C, LVDD, and serumγKlotho level are the independent risk factors that affect the long‐term prognostic outcome of MVD patients. SerumγKlotho level can effectively predict the long‐term prognosis of MVD patients. Based on our results, the risk of MACEs in MVD patients increases by 0.017 times with every one ng/mL increase in serum γ Klotho level.

Klotho codifies the new anti‐aging protein and is linked with different biological processes, especially human longevity [9]. Discovery of Klotho resulted in a renewed interest in that has dramatically helped to understand the aging process. The serum Klotho level within the human body declines after 40 years [28], and is detectable among those developing aging‐associated diseases like cancer, hypertension, and kidney diseases [29].

Until now, three Klotho subtypes have been detected in mice and human beings, namely, full‐length transmembrane Klotho, secreted Klotho, and shed Klotho [28]. Of them, secreted Klotho is predominantly found in humans, especially within the kidneys [30]. The Klotho family consists of three members and is encoded by three distinct genes [10, 11]: α‐Klotho, β‐Klotho, and γ‐Klotho.

Among them, α‐Klotho, β‐Klotho, and γ‐Klotho genes are located on chromosomes 13q12, 4p14, and 15q22.31, and include 6, 5, and 14 exons, respectively. The former two are upregulated within kidneys and enterohepatic tissues, while the latter shows a selective expression within mouse brown adipose tissues and eyes [31].

It is reported in a previous study that [32]: In patients with coronary artery disease, the gene expression level and serum level αKlotho are decreased. As to βKlotho, it is reported that [13]: serum βKlotho level is decreased in coronary artery disease patients than healthy controls, and it is negatively correlated with the severity of degree of coronary arteriostenosis.

As a member of the Klotho subfamily [33], γKlotho contains 567 amino acids and also belongs to the glycosyl hydrolase 1 family as a single‐pass membrane protein possessing 567 amino acids. Research on the γKlotho is scarce. γKlotho has been previously analyzed in three studies in areas like Breast [15], prostate [17], and bladder cancers [17].

Trošt et al reported that in triple negative breast cancers, high expression level of KLG correlates with poor disease progression and that in vitro analysis indicated that KLG was a necessary factor for cell survival; depletion of KLG resulted in cell cycle arrest, apoptosis, and lead better prognosis than a lower expression level of γKlotho [15]. Immunohistochemical analysis using prostate biopsy specimens revealed that patients with high KLG expression in primary prostate cancer tissue had a significantly poor prognosis for overall survival [17]. Namely the impact of γKlotho on the prognosis of various disease are not same in various research.

It is found that: increased oxidative stress in γKlotho‐depleted cells suggesting that γKlotho enables cancer cells to cope with an oxidative environment and that cells become dependent on its expression to maintain this survival advantage. These findings indicate that γKlotho might have a treating role dealing with oxidative stress [15]. In this study we found A higher serum γKlotho level indicates a poorer long‐term prognosis in MVD patients.

As for the mechanism on how higher serum γKlotho correlated with poor prognosis, We propose two possible hypotheses: First: we have to think about the function of γKlotho on human body, according to previous research γKlotho enables cancer cells to cope with an oxidative environment. γKlotho might have treating role to alter oxidative stress. [15] As for cardiomyopathy of MVC patients, there is myocardial hypoxia which produce reactive oxygen species (ROS), and result in oxidative stress, in this case, maybe cardiomyopathy needs a high level of γKlotho to cope with inoxidative stress, and high level of γ Klotho secreted to protect from injury which will be resulted from oxidative stress. Namely MCV maybe resulted from other traditional risk factors like hypertension、diabtes and unhealthy life style etc, to deal with the oxidative stress in MVC, body decreted high level of γKlotho. Namely, high level of serum γKlotho level only reflects the severity and poor prognosis of MVD, it might exert a therapeutic effect like brain natriuretic peptide (BNP). Second: Oxidative stress arises when excessive ROS generation overwhelms the intrinsic antioxidant defense system, leading to irreversible cellular damage [34] αKlotho mitigates myocardial injury by activating Nrf2/ARE‐mediated suppression of oxidative stress [35]. As an anti‐aging protein, high level of β‐klotho can decrease CHD risk and promoted its prognosis through various mechanisms, including antioxidation and anti‐autophagy [36]. Interestingly, we found increased expression of γKlotho accompanied with decreased expression of αKlotho and βKlotho in several other cancers [37]. Together, our results demonstrated a high level of γKlotho can result poor prognosis. Maybe the poor prognosis of MVD patients who has high level of γKlotho resulted from decreasing of αKlotho and βKlotho.

Recently, as artificial intelligence has rapidly developed, nomograms are now being used widely in medicine to predict prognosis [38]; nomogram not only simplifies the process of predicting the prognosis but also enhance the accuracy [39, 40]. Our single‐center follow‐up study established a novel nomogram with high clinical usefulness to predict the patient's prognosis. After validation, this nomogram exhibits a high discriminability ability, enhanced consistency between actual prognosis and model prediction value, and good clinical usefulness.

Our study had some limitations. In this study, serum γKlotho content was positively associated with the poor prognosis of MVD patients, but the specific mechanism is not too clear. Thus, there is an urgent need to clarify the mechanism that how a higher serum γKlotho level results in poor prognosis in our further research.

Conclusion: Serum γKlotho level is a novel biomarker for predicting long‐term prognostic outcome in MVD cases. Serum γKlotho level also shows a positive correlation with the poor prognosis of MVD patients, and the developed nomogram can more accurately predict long‐term prognosis and displays good clinical utility.

## Author Contributions

Tuersunjiang Naman contributed to writing the article, analysis of the results, funding acquisition. Yu‐ting Zhang contributed to the external validation. Ayinuer Maihemuti performed the data analysis and critical revision. Hui Cheng, Wen‐bo Fu, Xiao‐lin Yu, Zhao Wang were responsible for collecting data and acquisition of follow up data. Yi‐ning Yang and Zi‐tong Guo contributed to design and supervision of the research.

## Ethics Statement

This study complies with the Declaration of Helsinki, Every participant in this study signed the informed consent forms.

## Consent

The authors have nothing to report.

## Conflicts of Interest

The authors declare no conflicts of interest.

## Data Availability

The data that support the findings of this study are available from the corresponding author upon reasonable request.
